# Maternal mortality in Central Province, Kenya, 2009-2010

**DOI:** 10.11604/pamj.2014.17.201.3694

**Published:** 2014-03-13

**Authors:** Onesmus Maina Muchemi, Agnes Wangechi Gichogo

**Affiliations:** 1 Field Epidemiology and Laboratory Training Program (KENYA); 2 Ministry of Health, Kenya

**Keywords:** Maternal mortality, review, cause of death, Kenya

## Abstract

**Introduction:**

Maternal mortality for Kenya was 488/100,000 live births in 2009. Maternal mortality estimate for Central Province is unknown. We retrospectively reviewed data between 1st July 2009 and 30th June 2010 to estimate the hospital based maternal mortality ratio, characterize deaths by time, place and person and describe possible causes of deaths in Central province, Kenya.

**Methods:**

We abstracted data using a standard form from maternal death notification and review forms and the district reproductive health reports. Data was entered and analyzed using Microsoft Excel.

**Results:**

There were 89,512 live births and 111 deaths. The facility-based maternal mortality ratio was 124/100,000 live births. Seventy-three (66%) deaths had been audited. Thirty seven (33%) were aged 25 to 34 years. The mean age was 31years (±6). Thirty seven (33%) had a parity of less or equal to 2. Most case deaths (19%, n = 21) had attended 2 or less antenatal visits. The main gestation was below 37 weeks with 48% (n = 53). The main mode of delivery was vaginal (26%, n = 29). Majority (35%, n = 32) case deaths had delivered a live birth. Thirty seven (33%) mothers had been stable on admission. The main reason for admission waslabor with 12% (n = 13). Thirty-eight (34%) died within 24 hours after admission. Majority (27%, n = 30) were admitted antepartum but 39% (n = 43) were postpartum at the time of death. Thirty-five (32%) died of hemorrhage and 8(7%) Eclampsia.

**Conclusion:**

Maternal mortality is of public health importance in the region. Most deaths occurred within 24 hours after admission. Third delay was important. Bleeding and Eclampsia were the main causes of death. A third (34%) of notified deaths were not reviewed.

## Introduction

Maternal mortality is the death of a woman while pregnant or within 42 days of termination of pregnancy, irrespective of the duration and the site of the pregnancy, from any cause related to or aggravated by the pregnancy or its management but not from accidental or incidental causes. It is the leading cause of death among women of reproductive age.[1]

In 2010, the global maternal mortality ratio was estimated at 210 per 100,000 live births in 2010 and 480 per 100,000 in Africa whereas Kenya's mortality ratio was estimated at 360 per 100,000(1). Africa therefore shared the highest burden especially sub-Saharan Africa. Maternal mortality ratio for Kenya was estimated at 488 per 100,000 live births according to Kenya Demographic Health Survey of 2009 and these results indicate that maternal mortality remains high in Kenya [2]. The target for Kenya is to reduce it to 147 per 100,000 live births by 2015.

Globally, the most common causes of maternal morbidity and mortality are hemorrhage, infection, high blood pressure, unsafe abortion, and obstructed labor (WHO, 2013). The major causes of maternal mortality in Africa are hemorrhage (33.9%), sepsis/infections (9.7%), hypertensive disorders (9.1%), HIV/AIDS (6.2%), obstructed labor (4.1%), abortion (3.9%)and anemia (3.7%) [3]. Although reporting of the causes of maternal deaths is incomplete in the Health Management Information System (HMIS), the leading causes in Kenya appear to be antenatal and postpartum hemorrhage. Others include Eclampsia, sepsis, ruptured uterus, and obstructed labor.[2]

The fifth United Nations Millennium Development Goal (MDG) on maternal health aims to reduce maternal mortality by three-quarters between 1990 and 2015. [4] This goal still remains a challenge in Africa due to the inability to reliably measure levels and trends of maternal mortality using existing health information systems. Most deaths can be averted even in resource limited settings provided information necessary to monitor levels and trends and to guide interventions is strengthened.

A maternal death surveillance and response system can provide the essential information to measure and monitor maternal mortality at sub-national level and stimulate and guide actions to prevent future maternal deaths. The system has the potential to provide real-time, frequent monitoring of maternal mortality levels, trends, causes and circumstances surrounding the deaths provided attention is made to ensure completeness of reporting and data accuracy. The system would also strengthen civil registration and vital statistics system in the long term. However, this process requires technical innovations and financial resources [5]. In many sub-Saharan African countries however, these processes are not effective in monitoring maternal mortality and their contributing factors due to underreporting, incomplete data and lack of analysis and utilization of data generated by the system.

Maternal mortality ratio for Central province is unknown and accurate data to monitor progress in service delivery performance are scarce. There are no data on trends of maternal mortality in health institutions because of poor reporting rates by hospitals [6]. No study had been done in Central Province to determine maternal mortality and the circumstances surrounding maternal deaths. This justified the need to have a baseline data that could provide a reference for monitoring service delivery and future investigations on maternal mortality and its contributing factors in the region. The information derived from the findings could also be useful to health providers in adopting interventions and policies for the reduction of maternal mortality and improvement of maternal health services.

This investigation therefore aimed at estimating the hospital based maternal mortality ratio, characterizing the deaths by time, place and person and describing the causes of maternal mortality in Central province, Kenya. The purpose was to formulate recommendations to be shared with stakeholders in reproductive health so as to improve maternal death surveillance, reviews and notifications and maternal health care services in general for the achievement of the fifth millennium development goal by 2015.

## Methods

The review was carried out in Central Province, Kenya, in December 2012. Based on the national population census of 2009, the province had a total population of 4,383,743with an annual growth rate of 1.6%. The rate of skilled deliveries was 73.8%, family planning coverage of 66.7% and approximately 92.7% clients attending at least one antenatal visit. There were 24 hospitals, 81 health centres and 304 dispensaries. Approximately 89,512 live babies were born in health facilities during the study period.

We carried out a descriptive retrospective review of maternal deaths reported from health facilities between 1^st^ July 2009 and 30^th^ June 2010. The study population comprised of women who had given birth in health facilities in Central Province during the study period. Data was abstracted using a standard form from the Maternal Death Notification and Review forms, and the monthly district reproductive health reports.

Maternal death was defined as any death of a woman while pregnant or within 42 days of termination of pregnancy, from any cause related to or aggravated by the pregnancy or its management but not from accidental or incidental causes (ICD-10).

Maternal Death Review (MDR) or Audit was defined according to the *National Guidelines for Maternal and Perinatal Death Notification and Reviews* as the qualitative in-depth investigation of the causes of, and the circumstances surrounding maternal deaths with the purpose of tracing the path of the woman through the health care system and within the facility, to identify any avoidable remedial factors to improve maternal care in the future. The process is incomplete unless an attempt is made to respond to the findings with appropriate action. An audited maternal death was ascertained if a notified death was accompanied by a filled Maternal Death Review form.

We structured the abstraction form on a Ms. Excel spreadsheet with columns containing variables similar to the Maternal Death Notification and Review forms. The variables extracted included: age, parity, ANC attendance, gestation, mode of delivery, status of the baby at birth, mother's status on admission, reasons for admission, timing of death since admission, stage of pregnancy on admission, stage of pregnancy at death and the reported cause of death. Variables on the Maternal Death Notification and Review forms that were severely incomplete were excluded from analysis. These included marital status, educational level, details of antenatal care, delivery care and newborn care, interventions, any other contributing factors, comments on potential avoidable factors and action points.

We entered data on the spreadsheet for each month starting from 1st July 2009 to 30th June 2010 to make a line list of the cases. The dataset was examined for obvious errors by comparing the computer entries with those in the Maternal Death Notification and Review forms. We made corrections before onset of data analysis. We finally presented the information derived from the analysis using tables, frequencies, proportions, measures of central tendency and dispersion.

## Results

One hundred and eleven (111) deaths were reported from 1^st^ July 2009 to 30^th^ June 2010 among 89,512 live births. The reporting was aligned to the government fiscal year that runs from 1^st^ July to 30^th^ June the following year. Among the 111 deaths, only 73 (66%) had been audited. It is a requirement that after every occurrence of death, a maternal review committee audits the death within 7 days and develops action points to mitigate the gaps that may have resulted to the death.

Table 1 shows the maternal and obstetric characteristics for the 111 maternal deaths. The case deaths had a mean age of 31 years (±6) with age ranging from 18 to 51 years. Thirty seven (33%) of the maternal deaths occurred in women aged between 25 to 34 years. Thirty seven (33%) of the mothers had a parity of less or equal to 2. Most of the case deaths 21 (19%) had attended 2 or less antenatal visits. The gestation age was commonly less than 37 weeks, with a proportion of 48% (n = 53). The average gestation was 31 weeks with a range of 7-44 weeks. A high proportion (26%, n = 29) of the deliveries were vaginal. For majority (35%, n = 32) of the case deaths, the baby was live at birth. In thirty seven (33%) of the deaths, the mother had been stable at the time of admission. The main reason for admission was labor accounting for 13 (12%) of the women followed by both post-partum hemorrhage and pre-Eclampsia and Eclampsia at 9 (8%) respectively. Thirty-eight (34%) died within 24 hours after admission. Majority of the mothers (27%, n = 30) who died were admitted during the antepartum stage of pregnancy (which is the period before onset of labor), and at the time of death, majority (39%, n = 43) of them were in the postpartum stage of pregnancy (which is the period after delivery).

**Table 1:** Maternal and Obstetric characteristics of Maternal Deaths in Central Province, Kenya, from July 2009 to June 2010

Characteristic	Maternal DeathsN = 111 n (%)
**Age ( years)**	
<25	9(8)
25-34	37(33)
>34	19(17)
Missing data	46(41)
**Parity**	
≤para 2	37(33)
>para 2	30(27)
Missing data	44(40)
**Antenatal attendance**	
≤2 visits	21(19)
>2 visits	14(13)
Missing data	76(68)
**Gestation**	
<37 weeks	53(48)
37-42 weeks	11(10)
>42 weeks	1(1)
Missing data	46(41)
**Mode of delivery**	
Vaginal	29(26)
Caesarean section	25(23)
Antepartum	11(10)
Missing data	46(41)
**Status of newborn**	
Alive	35(32)
Stillbirth	14(13)
Unborn	10(9)
Early Neonatal Death	1(1)
Missing	51(46)
**Mother's status on admission**	
Stable	37(33)
Critically ill	23(21)
Dead on arrival	1(1)
Missing data	50(45)
**Reason for admission**	
Normal labor	13(12)
Postpartum Hemorrhage	9(8)
(Pre)Eclampsia	9(8)
Antepartum hemorrhage	5(5)
Abortion	4(4)
Others	27(24)
Missing data	44(40)
**Timing of death since admission**	
<24 hours	38(34)
≥24 hours	30(27)
Dead on Arrival	1(1)
Missing data	42(38)
**Stage of pregnancy on admission**	
Antepartum	30(27)
Intrapartum	17(15)
Postpartum	16(14)
Abortion	4(4)
Dead on Arrival	1(1)
Ectopic pregnancy	1(1)
Missing data	42(38)
**Stage of pregnancy at death**	
Postpartum	43(39)
Intrapartum	10(9)
Antepartum	9(8)
Abortion	2(2)
Ectopic pregnancy	1(1)
Missing data	46(41)

Pregnancy complications contributing to the maternal deaths in Central Province are shown in Table 2. Thirty-five (32%) of the deaths were due to hemorrhage, 8(7%) Eclampsia, cardiovascular disease 4(4%), amniotic embolism 4(4%), HIV/AIDS and infections contributed 3 (3%) each. The rest contributing 1 (1%) each were reported as having been caused by either cardiac arrest, disseminated intravascular coagulation (DIC) or the cause was unknown. It is important to note however, that there was a high proportion of missing information for all the parameters that were evaluated.

**Table 2:** Pregnancy complications for maternal deaths in Central Province, Kenya, from July 2009 to June 2010

Cause of death	Maternal Deaths N = 111 n (%)
Hemorrhage	35(32)
Eclampsia	8(7)
Amniotic embolism	4(4)
Cardiovascular disease	4(4)
HIV/AIDS	3(3)
Infections	3(3)
Disseminated Intravascular Coagulation	1(2)
Cardiac Arrest	1(2)
Unknown	1(2)
Missing data	49(44)

## Discussion

We documented a facility-based maternal mortality ratio of 124/100,000 which was lower than the national estimates, and also lower than the national targets of 2015. The mortality ratio was lower compared to the 190 per 100,000 live births reported in the study that reviewed data collected for the period between January 1981 and September 1988 in Thika, a constituent district of Central Province [7]. Auditing of deaths had been fairly done, with 34% of the maternal deaths having not been reviewed.

Majority of the maternal deaths reported had occurred within 24 hours after admission. This was consistent with findings from a retrospective study conducted in Rift Valley Provincial General Hospital where 109 deaths that were recorded between 1994 and 1998 were evaluated. The study had revealed that 53% of the deaths had occurred within 24 hours of admission^8^. This is a clear indication that most maternal deaths are of emergency nature.

The deaths were prevalent among age group 25 to 34 years. This is consistent with the findings from a study in Moi Teaching and Referral hospital where a retrospective audit of 150 maternal deaths that had been recorded from 2004 to 2011 revealed that majority of the deaths had occurred in women between 25 and 34 years [9]. Similarly, a study at Kenyatta National Hospital among 253 maternal deaths found two-thirds of the women to have been between 25 and 35 years of age [10]. The study by Juma et al, 2000, revealed that the deaths were mainly from the 14 to 24 years age group. The variation could be explained by differences in demographic and reproductive characteristics.

Thirty seven of the case deaths were less or equal to para 2. This was in agreement with findings in the study in Kenyatta National Hospital [10]. The highest proportion of mothers had attended two antenatal visits or less with slightly more having attended two visits and a fifth having not attended any visit. Majority had been less than 37 weeks gestation. This was observed in the study in Moi Teaching and Referral hospital [9].

Most case deaths were admitted during ante partum period. This does not concur with the findings in Moi Teaching and Referral Hospital where mothers were mainly admitted during the ante partum period [9]. The main reason for admission was labor followed by postpartum hemorrhage and pre-Eclampsia/Eclampsia and majority were admitted in stable condition. However, the highest proportion died after delivery. The main mode of delivery was spontaneous vertex delivery. For majority of the women, the baby was live at birth. The main causes of death were hemorrhage and Eclampsia, with hemorrhage contributing more than half the deaths. Others were cardiovascular disease, amniotic embolism, HIV/AIDS, infections, unsuccessful general anesthesia, cardiac arrest, disseminated intravascular coagulation and unknown causes. This did not concur with the findings from the study in Moi Teaching and referral hospital where Eclampsia was the leading pregnancy complication leading to death [9]. The findings were however consistent with those in the Kilifi study [11].

This review encountered a number of limitations which included using facility-based rather than community-based data which theoretically limits representativeness andgeneralizability. Other limitations included incompleteness of data on most of the variables that were reviewed. Despite these limitations, the findings of the investigation represent an approximate situation of maternal mortality, its causes and circumstances surrounding maternal deaths in Central Province, Kenya.

## Conclusion

The facility-based maternal mortality ratio was 124 per 100,000 live births. This was lower than the national average and also below the national target of year 2015. Most of the deaths reviewed were preventable. The most affected age group was between 25 and 34 years. The highest deaths occurred within 24 hours after admission. There were higher proportion of deaths among mothers with a parity of 2 or less, those who had attended 2 or less antenatal visits and those with less than 37 weeks gestation. The fact that most mothers were admitted during ante partum period, in normal labor and in stable condition indicates that there could be factors within health facilities (third delay) that make mothers particularly at risk in this region. This is contrary to the notion that the first and second delays are most important. Hemorrhage and Eclampsia were the main causes of maternal death.
